# Effect of fecal microbiota transplantation in children with autism spectrum disorder: A systematic review

**DOI:** 10.3389/fpsyt.2023.1123658

**Published:** 2023-03-02

**Authors:** Jing Zhang, Gang Zhu, Lin Wan, Yan Liang, Xinting Liu, Huimin Yan, Bo Zhang, Guang Yang

**Affiliations:** ^1^Senior Department of Pediatrics, The Seventh Medical Center of Chinese PLA General Hospital, Beijing, China; ^2^Department of Pediatrics, The First Medical Center of Chinese PLA General Hospital, Beijing, China; ^3^Medical School of Chinese PLA, Beijing, China; ^4^Department of Neurology, Boston Children's Hospital, Harvard Medical School, Boston, MA, United States; ^5^Biostatistics and Research Design Center, Institutional Centers for Clinical and Translational Research, Boston Children's Hospital, Harvard Medical School, Boston, MA, United States; ^6^Second School of Clinical Medicine, Southern Medical University, Guangzhou, China

**Keywords:** autism, autism spectrum disorder, microbiota-gut-brain axis, systematic review, meta-analysis, fecal microbiota transplantation (FMT)

## Abstract

**Background:**

Fecal microbiota transplantation (FMT) may be helpful in the treatment of autism spectrum disorder (ASD) as rebalancing the gut microbiome has been shown to potentially improve behavioral symptoms in children with ASD.

**Methods:**

This systematic review was conducted to assess the effect of FMT for children with ASD. The Embase, PubMed, Web of Science, and Cochrane Library databases were searched for articles published from inception to October 6, 2022. Two reviewers independently screened the identified records and undertook data extraction.

**Results:**

The search identified a total of five studies: two prospective open-label studies, two retrospective observational studies, and a case report; however, no randomized controlled trial was identified. All five studies reported a significant post-FMT-treatment improvement in neuropsychological assessment of ASD. The two prospective open-label studies suggested that the Autism Behavior Checklist (ABC) score, and the Social Responsiveness Scale (SRS) score at the posttreatment assessment decreased from the baseline (Wilcoxon signed-rank test; all *p* < 0.01]). The two retrospective observational studies suggested that FMT helped to improve the ASD symptoms. One observational study reported that the Childhood Autism Rating Scale (CARS) score and ABC score of the constipation group decreased from the baseline after the second course assessment (CARS [baseline: mean 35.25 ± standard deviation 4.36, second course: 32.5 ± 3.1, *p* = 0.015]; ABC [baseline: 56.21 ± 16.08, second course: 46.54 ± 16.54, *p* = 0.046]). Another observational study found that both ABC and CARS scores decreased as the number of FMT courses increased, and significant differences were found at the end of each course as compared with the baseline.

**Conclusion:**

Compared with the baseline, FMT significantly improved symptoms of autism in children with ASD in observational studies. However, rigorously designed randomized controlled clinical trials are needed to establish the safety and efficacy of FMT as a treatment for ASD.

## 1. Introduction

Autism spectrum disorder (ASD) is a common, highly heritable, and heterogeneous neurodevelopmental disorder that is characterized by a specific combination of impairments in social interactions and repetitive behaviors, highly restricted interests, and/or sensory behaviors since early life (1). The Global Burden of Diseases, Injuries, and Risk Factors Study 2019 reported a global prevalence of ASD of 0.37% (369.39/100,000), and epidemiological studies have suggested a higher prevalence in males than in females (estimated ratio >3:1) (2– 5). Although no curative treatment currently exists (6), cognitive behavioral therapy is an efficacious treatment for ASD in children (7).

The pathogenesis of ASD has not been clearly elucidated. However, studies suggested that the gut microbiota may influence core symptoms of ASD (e.g., social communication, restricted interests, repetitive behaviors, and differences in sensory function) and other associated symptoms through the microbial gut-brain axis (8, 9). Disorders of the gut microbiota in children with ASD and significant differences between individuals with ASD and neurotypical children exist (8, 10, 11), and gut microbiota intervention therapy may be helpful for ASD symptom improvement (12–14). Some studies (15, 16) have suggested that microbial therapeutic interventions, such as fecal microbiota transplant (FMT) treatment, antibiotics, and probiotics, are of the novel therapeutic strategies for improving ASD-associated symptoms. Gut microbiota has been emphasized as a likely new target for future therapeutic interventions for ASD.

FMT is the delivery of the extract of beneficial intestinal flora from a healthy donor to a patient's gastrointestinal (GI) tract to restore intestinal homeostasis in the recipient (17, 18). Clinical trials have established the efficacy and safety of FMT in various diseases, including inflammatory bowel disease, recurrent *Clostridioides difficile* infections (CDI), metabolic syndrome, diabetes, and some neuropsychiatric disorders (19–22). Previous research has indicated that gut microbiota may influence brain development and behavior through the neuroendocrine, neuroimmune and nervous systems (23). Studies have shown that fecal microbiota transplantation (FMT) can modulate intestinal flora and potentially alter neurological pathways. For example, Li et al. (24) showed that FMT reduced tic severity in a mouse model of Tourette's syndrome by modulating intestinal flora and promoted the secretion of serotonin. Goo et al. (25) suggested that FMT could be a potential treatment for cognitive deficits and social withdrawal symptoms that are observed among fragile X syndrome or ASD patients. The FMT was possibly attributable to the consequent increases in the *Akkermansia muciniphila* population and decreased levels of tumor necrosis factor alpha (TNF-α) that can cross the blood-brain barrier and ionized calcium-binding adaptor molecule 1. In addition, patients with ASD are often associated with disruption of the intestinal barrier. de Magistris et al. (26) suggested that a higher percentage of 36.7% among pediatric patients with ASD and their relatives (21.2%) had abnormal intestinal permeability, compared to control children (4.8%). Furthermore, gut microbiota may be able to influence brain through changes in the immune system. For example, cytokines, such as TNF-α, that are associated with lymphocytes and ASD, can bind to brain endothelial cells and induce an intracerebral immune response (27). Also, because FMT is a potential intervention to normalize microbial diversity and community structure, a human-mice interspecies study suggested (28) that FMT from healthy human donors shifted the gut microbial profile in a mouse model of ASD closer to that of healthy mice.

Despite the previous studies indicating that the FMT may be helpful in treating ASD, there is insufficient evidence to support FMT as a practical management option for ASD. This review was conducted to evaluate the literature and to conduct a meta-analysis to determine the effect of FMT for treating the core and associated symptoms of ASD in children.

## 2. Methods

Our review process precisely followed the Preferred Reporting Items for Systematic Reviews and Meta-Analyses (PRISMA) approach (29). The protocol of this meta-review is available online (CRD42022350481). The review only included previously published clinical studies and case reports and did not contain any new research results on human participants or animals.

### 2.1. Search strategies

A systematic literature search was conducted in Embase, PubMed, Web of Science, and Cochrane Library without any date restrictions until the last search on October 6, 2022. The following Medical Subject Heading [MeSH] terms were used, including the root term and text word: autism, autism spectrum disorder, fecal microbiota transplant, and fecal microbiota transfer therapy. The search strategy for each database can be found in Supplementary Table S1.

### 2.2. Study selection

Two reviewers independently screened the citations produced from the database searches for inclusion in this review. EndnoteX9 was used to retrieve and review titles and abstracts for initial investigations to determine the relevance and to remove duplicate literature. The same two reviewers screened the full texts of each included article to determine the final eligibility. Any disagreements were resolved through discussion between the two reviewers until a consensus was reached. All peer-reviewed original research was included.

### 2.3. Eligibility criteria

Studies were included for full review if they met the following criteria: randomized controlled trials, open-label studies, observational studies, prospective and retrospective cohort studies, case-cohort studies, and case reports; the subjects are children < 18 years with ASD; the study includes FMT as intervention; the control group can be no FMT or placebo; the included outcome variables were for the ASD rating scales. Studies were excluded if they met the following criteria: abstracts, letters to the editor, conference proceedings, and cost-effectiveness studies. Furthermore, a manual search of the reference lists to identify potentially relevant articles was undertaken to identify additional studies.

### 2.4. Data collection

Two independent reviewers extracted data. Data items included first author, year, country, trial design, number and characteristics of participants, type and aspects of intervention, outcome measures, and results (Table 1). Outcome data from primary analyses were extracted in all studies. Discrepancies between the reviewers were rechecked and discussed until a consensus was reached.

**Table 1:** Characteristics of the reviewed studies.

**Study, year**	**Country**	**Study design**	**Sample size (n [male/female])**	**ASD diagnostic tool**	**Age (range), years**	**Intervention types**	**Intervention details**	**Duration**	**Behavioral outcomes**	**Gastrointestinal outcomes**
Kang et al., 2017 (30)	USA	Prospective, open-label	18 (16/2)	ADI-R	10.8 ± 1.6	10-week FMT+8-week follow-up	Vancomycin: oral daily (days 1-14) Prilosec: oral daily (days 12–74) MoviPrep: The standard kit was used, with half the dosage administered at approximately 10 a.m. and the other half at 4 p.m., to cleanse the bowel of vancomycin and feces (Day 15) SHGM: On day 16, initial dose: 2.5 × 10^12^ cells/ day and maintenance dose: 2.5 × 10^9^ cells/day for 7 or 8 weeks	18 weeks	ADI-R, PGI-III, CARS, ABC, SRS, VABS-II, (relative to baseline)	GSRS, DSR
Li et al. (31)	China	Prospective open-label	40 (37/3)	ADI-R	8.03 ± 3.73 (3–17)	4-week FMT + 8-week follow-up	The participants received 2 L GOLYTELY (polyethylene glycol) the night before the transplantation the oral capsule and rectal administration groups received the same dose (approximately 2 × 10^14^ CFU per patient) once a week for 4 weeks	12 weeks	CARS, BC, SRS, (relative to baseline)	GSRS, DSR
Zhang et al. (32)	China	Retrospective observational	49 (41/8)	ADI-R, DSM-5	5.67 ± 3.08	Two consecutive WMT (2 months) + 8-week follow-up	The dose (120 mL/day for 6 consecutive days) was injected *via* transendoscopic enteral tubing or nasojejunal tube into the patient within 1 h	16 weeks	CARS, ABC, SDSC, (relative to baseline)	BSFS
Pan et al.2022 (33)	China	Retrospective observational	42 (34/8)	DSM-5	6.00 (3.75–8.25)	At least two consecutive WMTs (2 months)	The fecal suspension (approximately 5.0 × 10^13^ bacteria) was injected (60–90 mL/day for 6 consecutive days) through a transendoscopic tube. The interval between each treatment course was 1 month	At least 2 months.	CARS, ABC, SDSC, (relative to baseline)	BSFS
Huang et al., 2022 (34)	China	Case report	1 (1/0)	/	18	3-week FMT + 12-week follow-up	The patient received FMT once every other day *via* transendoscopic enteral tubing for 1 week, for a total of three cycles	15 weeks	HAMA, HAMD, SCL-90	BSFS

ADI-R, Autism Diagnostic Interview-Revised; DSM-5, Diagnostic and Statistical Manual of Mental Disorders; PGI-III, Parent Global Impressions-III; CARS, Childhood Autism Rating Scale; ABC, Autism Behavior Checklist; SRS, Social Responsiveness Scale; SDSC, Sleep Disturbance Scale for Children; HAMA, Hamilton Anxiety Scale; HAMD, Hamilton Depression Scale; SCL-90, Symptom Checklist 90; BSFS, Bristol Stool Form Scale; GSRS, Gastrointestinal Symptom Rating Scale; VABS-II, Vineland Adaptive Behavior Scale II; DSR, Daily Stool Recording; /, not mention; SHGM, standardized human gut microbiota; CFU, colony forming unit.

In the study conducted by Kang et al. (30), 18 children with ASD and 20 neurotypical children were enrolled. In the study conducted by Li et al. (31), 40 children with ASD and 16 neurotypical children were enrolled. In the two studies, only children with ASD received the FMT treatment; therefore, only the outcome data of children with ASD were included in the meta-analysis. Patients in the study conducted by Zhang et al. (32) received two courses of FMT, and we extracted the outcome data from the end of the second course of their patients for the meta-analysis. According to the Bristol Stool Form Scale (BSFS) and the Rome IV diagnostic criteria, when the BSFS score was <3, the patient was diagnosed with constipation. The patients with ASD were grouped into two groups: constipation group and control group, and patients in both the groups received FMT. Although Zhang et al. (32) reported the outcome data of the two subgroups separately, the means and standard deviations of the two subgroups could be combined and used to assess the effect of FMT on the relief of ASD symptoms in the meta-analysis. Pan et al. (33) only presented their primary analysis results in figures and did not provide any numerical results. Thus, their study was excluded from the meta-analysis. A patient aged 18 in the case report (34) was not included in the meta-analysis.

The primary outcome was changes in neuropsychological test scores among the ASD patients after FMT. Autistic symptoms were assessed using the Autism Behavior Checklist (ABC) [for the details of ABC, see (35–37)], the Childhood Autism Rating Scale (CARS) [for the details of CARS, see (38)], and the Social Responsiveness Scale (SRS) [for the details of SRS, see Cholemkery et al. (39)]. A summary of collected neuropsychological tests and the characteristics of each instrument of the tests are shown in Table 2.

**Table 2:** Summary of collected neuropsychological test scores in the systematical review and meta-analysis.

**Outcome**	**Study**	**Test**	**Description**
Neuropsychological meansures	Kang et al. (30) Li et al. (31) Zhang et al. (32)	Childhood autism rating scale	• Contains 15 items, each rated on a 4-point scale • Total scores: 0–60 • Higher scores indicate more serious illness (30–36, mild to moderate; >36, severe)
	Kang et al. (30) Li et al. (31) Zhang et al. (32)	Autism behavior checklist	• Contains 57 items to assess the severity of current ASD symptoms • Includes five common areas: stereotypy, irritability, lethargy, inappropriate speech, and hyperactivity • A score >53 points indicates a high possibility of ASD
	Kang et al. (30) Li et al. (31)	Social responsiveness scale	• Contains 65 items • Assesses social impairments, which are a core issue in autism, including social awareness, social information processing, capacity for reciprocal social communication, social anxiety/avoidance, and autistic preoccupations and traits

### 2.5. Meta-analysis and assessment of heterogeneity

Fixed-effects meta-analyses were performed using the Review Manager (Version 5.1). Mean differences (MDs) and their 95% confidence intervals (CIs) were estimated, with a negative MD indicating a beneficial effect of the intervention compared to the control group. The Q-statistic was derived, and the chi-square test was conducted for testing the interstudy heterogeneity in effect sizes. A *p*-value <0.10 indicated significant heterogeneity. Heterogeneity in effect sizes between studies was explored using *I*-square (*I*^2^) statistics. An *I*^2^ value <25, >25, >50, or >75% indicates low, moderate, substantial, or considerable heterogeneity, respectively. Forest plots were created to visualize the point estimates of the study effects and their CI. As no randomized controlled trials were included in this review and meta-analysis, no risk of bias assessment was performed.

## 3. Results

**Figure 1:**
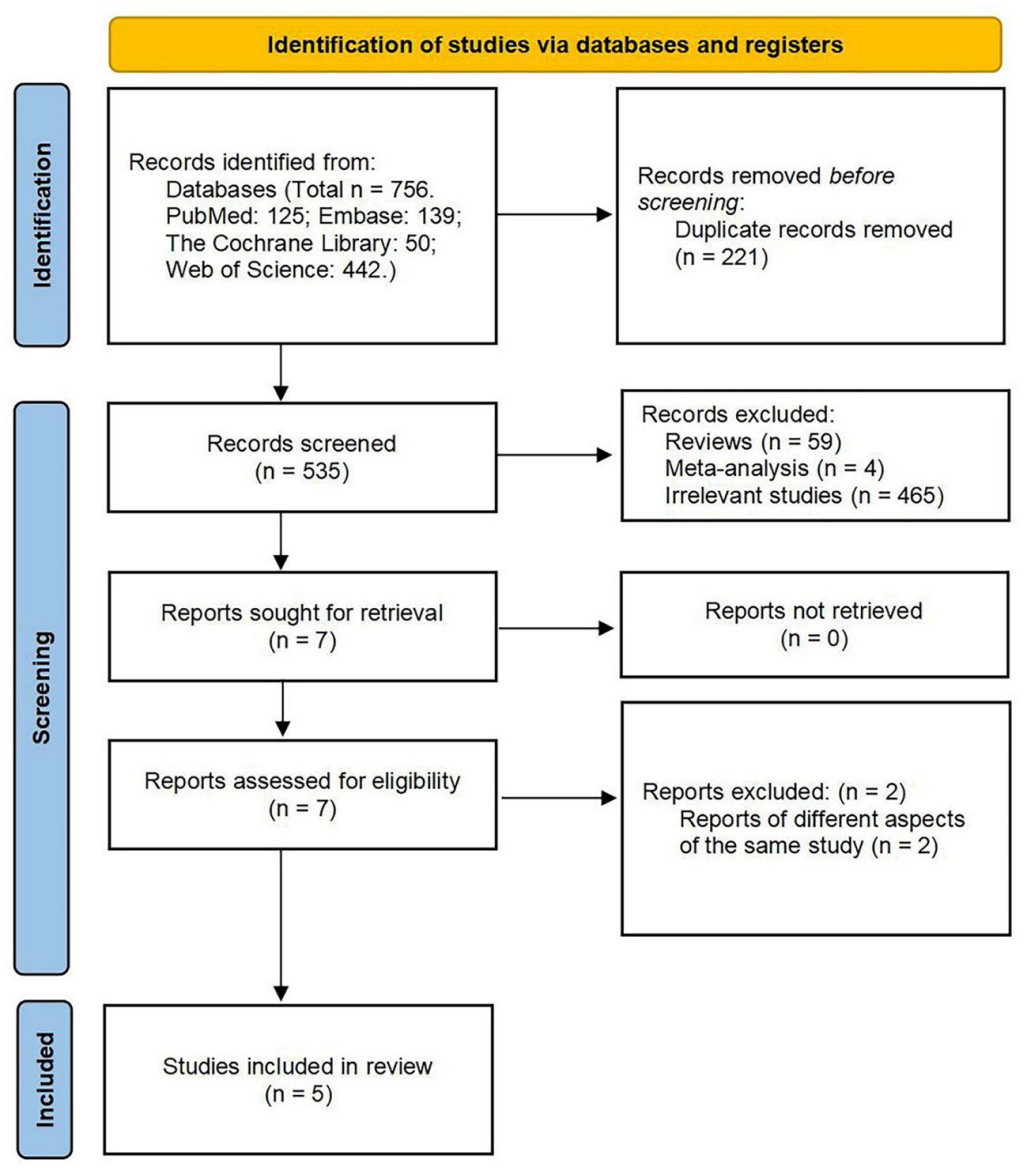
Preferred reporting items for systematic reviews and meta-analyses (PRISMA) 2020 flow diagram of the study selection process.

The database search identified a total of 756 potential references: 139 in Embase, 50 in the Cochrane Library, 125 in PubMed, and 442 in Web of Science. After de-duplication, a total of 535 articles were screened. The remaining seven articles were eligible for full-text review after scanning the titles and abstracts and removing the irrelevant literature and included two prospective open-label studies, two retrospective observational studies, and one case report that met the inclusion and exclusion criteria (Figure 1). The results of meta-analysis are reported in the Appendix.

### 3.1. Two prospective open-label studies

#### 3.1.1. Study characteristics

Table 1 presents the characteristics of included studies. The two prospective open-label studies were conducted, respectively in the United States [ClinicalTrials.gov, registration number NCT02504554 (30)] and in the People's Republic of China [China Clinical Trials Database, registration number: ChiCTR1800014745 (31)]. The study recruitment periods were not reported for these two studies. The participants were recruited from general hospitals in two major cities, Phoenix, Arizona, and Chongqing, People's Republic of China. All patients completed the entire course of the experiment without withdrawals (30, 31).

The Autism Diagnostic Interview-Revised (ADI-R) was used to diagnose ASD in two prospective open-label studies. The duration of FMT in the two studies was 10 weeks (30) and 4 weeks (31), respectively. The two studies were conducted among the patients aged 3 to 17 and were published in 2017 (30) and 2021 (31), respectively. The two studies (30, 31) provided information on the sex ratio of ASD subjects (mainly males) and noted at least one comorbidity, especially GI symptoms.

#### 3.1.2. Intervention characteristics

The two prospective open-label studies differed with regard to the type, procedure, and duration of FMT intervention. The treatment conducted by Kang et al. (30) commenced with 14 days of oral vancomycin. Prilosec was administered starting on the 12th day of the vancomycin treatment course and was continued until the end of the lower dosage of standardized human gut microbiota (SHGM). On Day 15, the patients were administered MoviPrep (containing mainly polyethylene glycol) to remove most of the remaining gut bacteria and vancomycin. On Day 16, the participants received either oral or rectal SHGM at two different doses: the high major dose and a lower maintenance dose. The high-dose SHGM regimen comprised a daily dosage of 2.5 × 10^12^ cells, with 2 days of oral and 1 day of rectal SHGM administration. A maintenance dose of 2.5 × 10^9^ cells/day SHGM was administered orally for the following 7–8 weeks. Li et al. (31) did not administer antibiotic and proton pump inhibitor therapy before FMT. All FMT participants were administered 2 L polyethylene glycol before FMT. The same dose (approximately 2 × 10^14^ colony forming unit (CFU)/person) was used for both oral capsule and rectal administration groups once a week for 4 weeks. The intervention was performed by a clinician or professional, and the summary judgment of each score was undertaken by a pediatrician or qualified professional (31).

During the specific implementation of FMT, the patients in the study conducted by Li et al. (31) did not receive vancomycin and proton pump inhibitors before the start of FMT, and thus ruled out any interference of vancomycin and proton pump inhibitors in the effect of FMT. The stool samples used for FMT were subjected to rigorous screening and preparation to ensure the safety of FMT. Both trials were unblinded to the investigators. Patients could choose the oral or rectal route for flora transplantation depending on whether they could swallow the capsule or had a strong personal preference. The study found no statistical difference in the effect of the oral and rectal routes of FMT (30, 31).

#### 3.1.3. Characteristics of outcome measures and investigation results

The ASD symptoms were the primary study endpoints in a prospective open-label study (31) and were the secondary endpoint in the other prospective open-label study (30). The number of participants with outcomes analyzed comprised 47% (18/38 subjects) (30) and 71% (40/56 subjects) of the cohorts (31). For the 2 studies with a specified follow-up period, the treatment effects on ASD symptoms were maintained at the 8-week follow-up (30, 31).

ASD symptoms were assessed using the CARS, ABC, SRS scores, and the Vineland Adaptive Behavior Scale II (VABS-II). Kang et al. (30) suggested that the CARS score at the posttreatment assessment (mean ± standard deviation: 34.06 ± 4.56) significantly decreased from the baseline (39.67 ± 5.30, *p* < 0.001, obtained from a Wilcoxon signed-rank test). The CARS score at the 8-week follow-up (33.69 ± 5.38) also significantly decreased from that of the baseline (39.67 ± 5.3, *p* < 0.001). The SRS score at the post-treatment (97.78 ± 27.13) assessment had substantially decreased from baseline (116.17 ± 24.16, *p* < 0.001). The ABC score at the post-treatment assessment (47.75 ± 32.33) significantly decreased from baseline (63.58 ± 29.95, *p* < 0.01). The ABC score of 8-week follow-up (47.86 ± 27.92) were significantly decreased from baseline (63.58 ± 29.95, *p* < 0.01). The VABS-II scoring evaluates adaptive behaviors and their association with age, intelligence quotient, and autistic symptoms for a functionally heterogeneous. The average developmental age increased by 1.4 years (*p* < 0.001); however, the final VABS-II age equivalent was still lower than their chronological age. Kang et al. (40) followed up the 18 patients for 2 years and found that their ASD symptoms improved at the 2-year follow-up. Li et al. (31) showed that FMT helped to improve the ASD symptoms. Following FMT treatment, the ABC score decreased significantly (mean±standard deviation of baseline: 86.08 ± 21.45, post-treatment: 67.25 ± 19.01, *p* < 0.001, obtained from a Wilcoxon signed-rank test), and there was no apparent reversion during 8-week follow-up (73.67 ± 18.23) compared with that at the post-treatment assessment (67.25 ± 19.01, *p* > 0.05). The CARS score at the post-treatment assessment decreased significantly relative to that at the baseline (baseline: 37.55 ± 7.16, post-treatment: 28.25 ± 6.15, *p* < 0.0001). Furthermore, the improvements of the SRS score were reversed after 8 to 12 weeks without a further course of FMT (31).

### 3.2. Two retrospective observational studies

#### 3.2.1. Study characteristics

Table 1 lists the characteristics of included studies. Two retrospective observational studies were conducted in the People's Republic of China and were published in 2022. One study was registered in the Chinese clinical trials database [registration number: ChiCTR2100044807 (32)]. These two observational studies reported the recruitment period [June 2019 to July 2021 (32) and June 1, 2019, to June 30, 2021 (33)]. The study participants were recruited from general hospitals in Guangdong, People's Republic of China. All patients completed the entire course of FMT without withdrawals.

The ADI-R (32) and Fifth Edition of the Diagnostic and Statistical Manual of Mental Disorders (DSM-5) (32, 33) were used to diagnose ASD. The duration of FMT was 2 months. The median age of the patients in the study conducted by Pan et al. (33) was 6.00 years (interquartile range: 3.75–8.25), and the sample size was 42. The age range of the patients in the study conducted by Zhang et al. (32) was 3–14 years, and the sample size was 49. The studies provided information on the sex ratio of ASD subjects (mainly males) and included at least one comorbidity in the ASD patients in the two studies, particularly sleep disorders and GI symptoms (32, 33).

#### 3.2.2. Intervention characteristics

The two retrospective observational studies differed with regard to the type and duration of FMT intervention. Pan et al. (33) injected fecal suspension (approximately 5.0 × 10^13^ bacteria) *via* an endoscopic bowel tube (60–90 mL/day for six consecutive days) and evaluated before each injection. Each patient underwent at least two courses of FMT treatment, with a final evaluation at 1 month after the end of the last course. Zhang et al. (32) used transendoscopic enteral tubing (lower digestive tract) or nasojejunal tube (middle digestive tract; 120 mL/day for six consecutive days) to inject fecal suspension for 4 weeks in two treatment courses. The intervention was performed by a clinician or professional, and the summary judgment of each scale score was undertaken by a pediatrician or qualified professional.

#### 3.2.3. Characteristics of outcome measures and investigation results

Improvement of ASD symptoms was the primary study endpoint in 1 retrospective observational study (33) and the secondary endpoint in the other (32), as assessed by both CARS and ABC (32, 33). The number of participants with outcomes analyzed in the meta-analyses was 100% (49/49 subjects) (32) and 0% (0/42 subjects) (33). For these two retrospective observational studies with a specified follow-up period, treatment effects on ASD symptoms were assessed at 2 months (32, 33).

Zhang et al. (32) reported that the CARS score of the constipation group at the end of the second course (mean ± standard deviation: 32.5 ± 3.1) had significantly decreased from baseline (35.25 ± 4.36, *p* = 0.015, obtained from a Wilcoxon signed-rank test). The CARS score of the control group after the first course (34.54 ± 3.37), was statistically significant from the baseline (36.64 ± 3.38, *p* = 0.033). Furthermore, the CARS score of the control group after the second course (33.88 ± 2.61) was significant decreased from the baseline (36.64 ± 3.38, *p* = 0.002). The ABC score of the constipation group after the second course (46.54 ± 16.54) differed significantly from the baseline (56.21 ± 16.08), *p* = 0.046. Pan et al. (33) found that both ABC and CARS scores decreased as the number of FMT courses increased, and significant differences were found at the end of each course as compared with the baseline. More courses led to significantly lower ABC scores [second FMT vs. first FMT: −6.50 (interquartile range:−19.00, −2.00) vs. −5.00 (−10.50, 2.25), *p* = 0.045; third FMT vs. second FMT: −14.04±16.62 vs. −8.83±13.96, *p* = 0.022]. No statistically significant difference was observed in the fourth and fifth courses compared to the previous adjacent courses. The CARS scores gradually decreased with additional FMT courses, but there was no significant difference between the two adjacent courses (33).

### 3.3. A case report

Asperger syndrome (AS) was described as the behavioral characterization of individuals with difficulties in communication and social interaction (40). The diagnostic labeling of AS was removed from DSM-5 and AS was listed under a more general category of ASD (41, 42). The first case of FMT in an adult patient with AS was reported by Huang et al. (34) in 2022 as an 18-year-old man diagnosed with AS 12 years ago but without a description of the diagnostic criteria. The patient suffered from diarrheal irritable bowel syndrome for 6 years, with diarrhea and abdominal pain as the main symptoms. The details of FMT were: 150 mL of saline containing 50 cm^3^ of centrifugal flora was administered through an endoscopic enteral catheter 3 times a week for 3 consecutive weeks. The intervention was performed by a clinician or professional, and the summary assessment of each scale score was done by a pediatrician or a qualified professional. After three rounds of FMT, AS symptoms improved significantly. The treatment effect persisted at 1 month follow-up. Still, the patient's psychological condition reappeared in the 3rd month after FMT, possibly related to mood swings caused by a breakup with his girlfriend in the 2nd month after FMT. FMT may alleviate AS by restoring the gut microbiota, primarily by increasing the number of bacteria associated with short-chain fatty acids. The gut microbiota changed after FMT, but regression occurred at 3 months after FMT. Huang et al. (34) also identified some metabolites changed after FMT but did not provide any evidence on association of the changes with the ASD symptom improvement.

### 3.4. Effect of FMT on other ASD-associated symptoms

#### 3.4.1. Improvement of GI symptoms

The GI outcomes were determined using the BSFS, Gastrointestinal Symptom Rating Scale (GSRS) and Daily Stool Recording (DSR). Kang et al. (30) showed a reduction in GI symptoms of ~80% at the end of FMT treatment, including significant improvement in constipation, diarrhea, dyspepsia, and abdominal pain. The DSR showed a significant decrease in days with abnormal or no bowel movement (Wilcoxon signed-rank test: *p* = 0.002) and these improvements were maintained after 8 weeks of no treatment. Li et al. (31) found that children with ASD had a 35% decrease in mean GSRS score after the treatment for 8 weeks, indicating significant improvement of GI symptoms. The presence of no stools, hard stools (type 1 or 2), and soft/liquid stools (type 6 or 7) was significantly reduced at the end of FMT treatment, and these effects were maintained at 8 weeks after FMT. Pan et al. (33) found that FMT significantly improved GI symptoms in children with ASD. Zhang et al. (32) found that FMT relieved constipation in children with ASD without worsening stool morphology in non-constipated children with ASD.

#### 3.4.2. Improvement of sleeping disorders

Sleep disturbance-related outcomes were determined using the Sleep Disturbance Scale for Children (SDSC). Both the retrospective observational studies indicated that FMT could reduce the SDSC scores and improve sleep disturbances in children with ASD. Pan et al. (33) found that more courses of FMT resulted in a significant reduction in the SDSC scores, and patients who received three courses of FMT resulted in more substantial improvement in sleep disturbances.

#### 3.4.3. Intestinal flora changes after FMT

The two prospective open-label studies (30, 31) showed that the gut microbiota after FMT more closely resembled that of neurotypical children and donors. Kang et al. (30) detected several beneficial changes in the intestinal environment. Specifically, overall bacterial diversity and the abundances of *Bifidobacterium, Prevotella, Desulfovibrio*, and other taxa increased after FMT, and these changes persisted after an 8-week untreated period. Both *Prevotella* and *Desulfovibrio* populations were more abundant in FMT recipients than in donor samples (30). Li et al. (31) found that the abundance of *Eubacterium coprostanoligenes* and the GSRs scores before FMT were positively correlated and he reduction in the *Eubacterium coprostanoligenes* population was positively associated with a reduction in GI symptoms. Huang et al. (34) found that FMT could alleviate AS by restoring and improving the gut microbiota structure, mainly by increasing the abundance of bacteria associated with short-chain fatty acids, such as *Roseburia, Bifidobacterium, Ruminococcus, Prevotella, and Faecalibacterium*. As the stool donors varied across studies and individual variations, an accurate conclusion about bacterial changes after FMT could not be ascertained.

### 3.5. Adverse effects of FMT on ASD

Two studies recorded and judged the adverse effects that occurred with the FMT treatment. Kang et al. (30) found no severe adverse reactions during and after the intervention, and that mild to moderate adverse reactions were mainly caused by vancomycin, such as a rash (5%), hyperactivity (39%), tantrums/aggression (28%), and nausea/vomiting (high-dose SHGM: 5%). Li et al. (31) found that FMT caused minimal side effects, including hyperactivity and aggression, and was well-tolerated.

## 4. Discussion

ASD is the most common, typical neurodevelopmental disorder that impairs social interactions and communication and leads to restricted, repetitive, and stereotypical patterns of behavior, interests, and activities (1). The pathogenesis of ASD is unclear, and an increasing number of studies have shown that ASD is caused by the interaction of genetic and environmental factors. The microbiota-gut-brain axis plays an important role in ASD (8, 9). Many children with ASD experience significant GI symptoms, such as constipation, diarrhea, and alternating constipation/diarrhea, which correlate with ASD severity. Such GI symptoms appear to be due, in part, to dysbiotic gut microbiota and perhaps their missing roles in the modulation of metabolites that affect GI function and neurobiological conditions, such as ASD and anxiety (43). There are significant differences in the intestinal microbiota profiles of children with ASD and neurotypical children (44). In children with ASD, a small open-label study found that 8 weeks of treatment with oral vancomycin (a non-absorbable antibiotic which acts only in the gut) led to major improvements in both GI symptoms and ASD symptoms, although the benefits were lost within a few weeks after treatment was stopped (30).

FMT, also known as fecal microbiota transfer therapy, refers to the transfer of intestinal flora from healthy donors into the intestines of patients by gastroenteroscopy, capsules, enema, etc., to reconstruct the intestinal homeostasis of patients to achieve the purpose of treatment (17, 18). Clinical studies have reported the efficacy and safety of FMT in the treatment of a variety of diseases, including inflammatory bowel disease, CDI, metabolic syndrome, and some neuropsychiatric diseases (19–22). Accumulating evidence has strengthened a link between dysbiotic gut microbiota and autism (25, 28). FMT is a promising therapy to repair dysbiotic gut microbiota of ASD. However, the evidence is insufficient to support FMT as a practical approach for ASD.

We reviewed the existing literature and conducted a meta-analysis to investigate the potential effect of FMT for treating the core and associated symptoms of children with ASD. We searched the Embase, PubMed, Web of Science, and Cochrane Library and reviewed the studies published previously to assess the effect of FMT in improving autistic symptoms in children with ASD. This review included two prospective open-label studies, two retrospective observational studies, and a case report. The five studies reported a significant improvement in core and associated symptoms of ASD after the FMT and until the end of follow-up. No severe adverse effects were reported in the studies, suggesting the FMT is a safe treatment approach for patients with ASD. Three studies were included in the meta-analyses (30–32). Compared with the baseline, FMT was associated with a significant reduction in CARS, ABC, and SRS, which showed FMT may improve the core symptoms of ASD. However, the reviewed studies are either observational studies or a case report. Therefore, the effect of the FMT is confounded with other factors and its efficacy cannot be determined as in randomized controlled trials. Although FMT may have some short-term beneficial effects, we did not find high-quality evidence of long-term benefits associated with FMT.

FMT significantly improved GI symptoms in children with ASD and relieved constipation in children with ASD without worsening stool morphology in non-constipated children with ASD. Approximately 2/3 of children with ASD have sleep disturbance which can lead to extreme daytime behavior in children with ASD (45), and even some children need sleep pills or tranquilizers. Families are reluctant to put patients on long-term medication for safety reasons. Both 2 retrospective observational studies indicated that FMT could reduce SDSC scores and improve sleep disturbances in children with ASD and more courses of FMT resulted in a significant reduction in SDSC scores. Therefore, FMT may be an effective treatment for sleep disorders in children with ASD and a substitute for psychotropic drugs.

Intestinal flora of patients with ASD obviously changed after FMT. The two prospective open-label studies (30, 31) showed that after FMT the condition of gut microbiota of the ASD patients was close to that of neurotypical children and donors. These results suggested that FMT may bring the intestinal flora of children with ASD closer to the donor flora and increase the abundance of beneficial flora. Kang et al. (30) found overall bacterial diversity and abundance of *Bifidobacterium, Prevotella, Desulfovibrio*, and other taxa increased after FMT, and these changes persisted after an 8-week untreated follow-up. Both *Prevotella* and *Desulfovibrio* were more abundant in FMT recipients than in donor samples, indicating that the transferred microbiota recruited new symbiotic bacteria in a friendly manner to modify the intestinal environment (30).

With the advancement in technology, the way of selecting suitable donors, preparing the digestive system, and delivering FMT has greatly improved. A variety of advanced therapeutic modalities had been made available to treat various diseases. These advanced therapeutic modalities of FMT include washed microbiota transplantation for inflammatory bowel disease (46), colonic transendoscopic enteral tubing for the delivery of washed microbiota transplantation (47), and prebiotics-encapsulated probiotic spores for cancer treatment (48) and the microbiota suspension for recurrent clostridium difficile infection (49).

Preselection of FMT donors based on the presence of specific microbiota strains is a promising approach to improve clinical outcomes and is being developed toward a precision treatment (50). However, there are several challenges in taking FMT from the laboratory to the clinic. Encouragingly, the safety and effectiveness of FMT in treating CDI have been confirmed by two randomized, double-blind, placebo-controlled clinical studies (51, 52). As a result, the United States Food and Drug Administration has approved FMT as a treatment to prevent CDI recurrence in adults (https://www.fda.gov/news-events/press-announcements/fda-approves-first-fecal-microbiota-product), which will accelerate its translation from the laboratory to the clinic and increase its use as a treatment.

It should be noted that there are several limitations to our study. The main limitation is that all of the studies included in our systematic review and meta-analyses were non-randomized, non-blinded, and there were only a few studies included. Therefore, we must exercise caution in interpreting the precise quantitative estimates of the effects of FMT on children with ASD. Despite these limitations, our meta-analyses do provide valuable insight into the potential benefits of FMT for children with ASD. Yet, due to the limitations of the data, we have rated the certainty of the treatment's effect as low.

## 5. Conclusion

In conclusion, this review showed that, in the observational and case studies, the FMT showed its potential in reducing the CARS, ABC, and SRS scores and improving ASD symptoms among children with ASD. FMT may be a potential therapy for alleviating symptoms of ASD in children with ASD. However, rigorously designed randomized double-blind placebo-controlled trials are needed to establish the safety and efficacy of FMT as a treatment to ASD.

## Data availability statement

The original contributions presented in the study are included in the article/Supplementary material, further inquiries can be directed to the corresponding author.

## Author contributions

Protocol was designed by JZ, XL, and HY. Search was performed by JZ, and reference screening undertaken by GZ. Data extraction was performed by JZ and double-checked by GZ and LW. The manuscript was written by JZ, GZ, and LW and reviewed by BZ and GY. All authors contributed to the article and approved the submitted version.

## Figures and Tables

**Figure A1:**
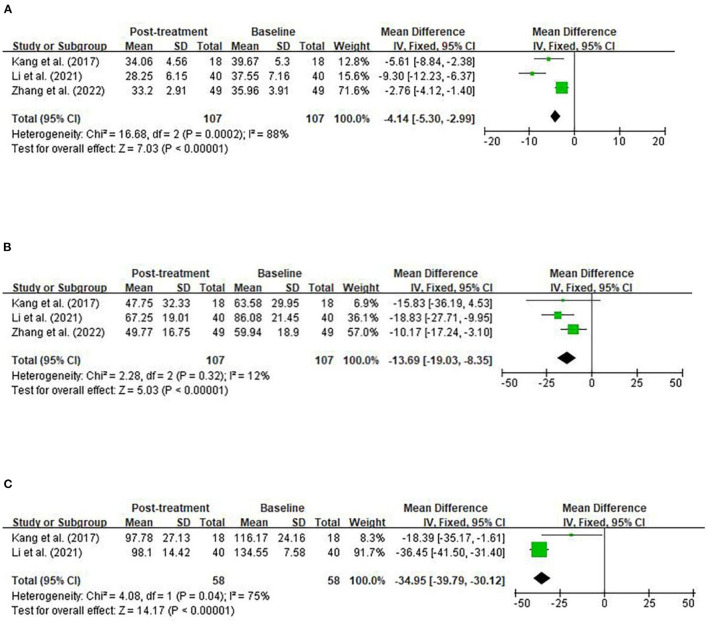
Fixed-effects meta-analyses of parental intervention on CARS **(A)**, ABC **(B)**, and SRS **(C)**. CARS, Childhood Autism Rating Scale; ABC, Autism Behavior Checklist; SRS, Social Responsiveness Scale; SD, standard deviation; CI, confidence interval; d.f., degrees of freedom; *I*^2^, *I*-square; *Z*, z-statistic.

## Appendix

### Meta-analysis

No randomized trials were identified of any interventions that directly addressed the targeted study populations. Neuropsychological testing in three studies evaluated the CARS and ABC score (30–32) and two studies evaluated the SRS score (30, 31) to assess the improvement of autism-related symptoms. Figure A1A shows the results of the meta-analysis on the effect of the FMT on the CARS score. Compared with the baseline, FMT was associated with a significant reduction in the CARS score. The meta-analysis was conducted using a fixed-effects model for the two prospective open-label studies and one retrospective observational study (30–32). The mean difference (MD) was −4.14 on a numerical rating scale (95% CI: [−5.30, −2.99], *z*-statistic = 7.03, *p* < 0.0001) and the heterogeneity chi-squared statistic was 16.68 (degrees of freedom [d.f.] = 2, *p* = 0.0002, *I*^2^=88%). Figure A1B shows the results of the meta-analysis of the effect of the FMT on the ABC score. Compared with the baseline, FMT induced a significant reduction in the ABC score. The meta-analysis was conducted for the 2 prospective open-label studies and 1 retrospective observational study (30–32). The MD was −13.69 (95% CI: [−19.03, −8.35], *z*= 5.03, *p* < 0.0001). The heterogeneity chi-square statistic was 2.28 (d.f.=2, *p*=0.32; *I*^2^=12%). Figure A1C shows the results of the meta-analysis of the effect of the FMT on the SRS score using a fixed-effects model. Compared with the baseline, FMT was associated with a significant reduction in the SRS score [2 prospective open-label studies (30, 31), MD, −34.95 on a numerical rating scale (95% CI: [−39.79, −30.12], *z*=14.17, *p* < 0.00001. The heterogeneity chi-square statistic was 4.08 (d.f. = 1, *p* = 0.04; *I*^2^=75%). Heterogeneity was high in the meta-analyses on the CARS and SRS scores and low in the meta-analysis on the ABC score.
