# Supplementary material for: Effect of fecal microbiota transplantation in children with autism spectrum disorder: A systematic review
Source: Front Psychiatry. 2023 Mar 2;14:1123658. doi: 10.3389/fpsyt.2023.1123658 (PMC10017995; doi:10.3389/fpsyt.2023.1123658)
Supplement: Supplementary file 1 [file Table_1.DOCX]

Supplementary Material

- Effect of Fecal Microbiota Transplantation in Children With Autism Spectrum Disorder: A Systematic Review and Meta-analysis

**Jing Zhang^1,2,3#^, Gang Zhu^1,2,3#^, Lin Wan^1,2,3#^, Yan Liang^1,2,3^, Xinting Liu^1,2,3^, Huimin Yan^1,2,3^, Bo Zhang^4,5*^, Guang Yang^1,2,3,6*^**

*** Correspondence:**Dr. Guang Yang

[yangg301@126.com](mailto:yangg301@sina.com)

Dr. Bo Zhang

[bo.zhang@childrens.harvard.edu](mailto:bo.zhang@childrens.harvard.edu)

# Supplementary Table

**Table S1. Search strategies in the selected databases**

| Embase | ('autism spectrum disorder':ab,ti OR 'autism spectrum disorders':ab,ti OR 'autistic spectrum disorder':ab,ti OR 'autistic spectrum disorders':ab,ti OR 'disorder, autistic spectrum':ab,ti OR 'syndrome, asperger':ab,ti OR 'aspergers disease':ab,ti OR 'aspergers diseases':ab,ti OR 'disease, aspergers':ab,ti OR 'diseases, aspergers':ab,ti OR 'asperger disease':ab,ti OR 'asperger diseases':ab,ti OR 'disease, asperger':ab,ti OR 'diseases, asperger':ab,ti OR 'asperger disorders':ab,ti OR 'disorder, asperger':ab,ti OR 'disorders, asperger':ab,ti OR 'aspergers disorder':ab,ti OR 'asperger disorder':ab,ti OR 'disorder, aspergers':ab,ti OR 'aspergers syndrome':ab,ti OR 'asperger syndrome':ab,ti OR 'syndrome, aspergers':ab,ti OR 'autistic disorder':ab,ti OR 'disorder, autistic':ab,ti OR 'disorders, autistic':ab,ti OR 'kenners syndrome':ab,ti OR 'kenner syndrome':ab,ti OR 'kanters syndrome':ab,ti OR 'autism, infantile':ab,ti OR 'infantile autism':ab,ti OR autism:ab,ti OR 'autism, early infantile':ab,ti OR 'early infantile autism':ab,ti OR 'infantile autism, early':ab,ti) AND ('fecal microbiota transplantation':ab,ti OR 'fecal microbiota transplantations':ab,ti OR 'microbiota transplantation, fecal':ab,ti OR 'microbiota transplantations, fecal':ab,ti OR 'transplantation, fecal microbiota':ab,ti OR 'transplantations, fecal microbiota':ab,ti OR 'fecal microbiota transplant':ab,ti OR 'fecal microbiota transplants':ab,ti OR 'microbiota transplant, fecal':ab,ti OR 'microbiota transplants, fecal':ab,ti OR 'transplant, fecal microbiota':ab,ti OR 'transplants, fecal microbiota':ab,ti OR 'fecal microbiome transplantation':ab,ti OR 'fecal microbiome transplantations':ab,ti OR 'microbiome transplantation, fecal':ab,ti OR 'microbiome transplantations, fecal':ab,ti OR 'transplantation, fecal microbiome':ab,ti OR 'transplantations, fecal microbiome':ab,ti OR 'fecal transplant':ab,ti OR 'fecal transplants':ab,ti OR 'transplant, fecal':ab,ti OR 'transplants, fecal':ab,ti OR 'donor feces infusion':ab,ti OR 'donor feces infusions':ab,ti OR 'feces infusion, donor':ab,ti OR 'feces infusions, donor':ab,ti OR 'infusion, donor feces':ab,ti OR 'infusions, donor feces':ab,ti OR 'intestinal microbiome transplant':ab,ti OR 'intestinal microbiome transplants':ab,ti OR 'microbiome transplant, intestinal':ab,ti OR 'microbiome transplants, intestinal':ab,ti OR 'transplant, intestinal microbiome':ab,ti OR 'transplants, intestinal microbiome':ab,ti OR 'intestinal microbiota transfer':ab,ti OR 'intestinal microbiota transfers':ab,ti OR 'microbiota transfer, intestinal':ab,ti OR 'microbiota transfers, intestinal':ab,ti OR 'transfer, intestinal microbiota':ab,ti OR 'transfers, intestinal microbiota':ab,ti OR 'intestinal microbiota transplantation':ab,ti OR 'intestinal microbiota transplantations':ab,ti OR 'microbiota transplantation, intestinal':ab,ti OR 'microbiota transplantations, intestinal':ab,ti OR 'transplantation, intestinal microbiota':ab,ti OR 'transplantations, intestinal microbiota':ab,ti OR 'intestinal microbiome transplantation':ab,ti OR 'intestinal microbiome transplantations':ab,ti OR 'microbiome transplantation, intestinal':ab,ti OR 'microbiome transplantations, intestinal':ab,ti OR 'transplantation, intestinal microbiome':ab,ti OR 'transplantations, intestinal microbiome':ab,ti OR 'intestinal microbiota transplant':ab,ti OR 'intestinal microbiota transplants':ab,ti OR 'microbiota transplant, intestinal':ab,ti OR microbiota:ab,ti OR 'transplants, intestinal':ab,ti OR 'transplant, intestinal microbiota':ab,ti OR 'transplants, intestinal microbiota':ab,ti OR 'intestinal microbiome transfer':ab,ti OR 'intestinal microbiome transfers':ab,ti OR 'microbiome transfer, intestinal':ab,ti OR 'microbiome transfers, intestinal':ab,ti OR 'transfer, intestinal microbiome':ab,ti OR 'transfers, intestinal microbiome':ab,ti OR 'fecal microbiota transfer':ab,ti OR 'fecal microbiota transfers':ab,ti OR 'microbiota transfer, fecal':ab,ti OR 'microbiota transfers, fecal':ab,ti OR 'transfer, fecal microbiota':ab,ti OR 'transfers, fecal microbiota':ab,ti OR 'fecal transplantation':ab,ti OR 'fecal transplantations':ab,ti OR 'transplantation, fecal':ab,ti OR 'transplantations, fecal':ab,ti OR 'washed microbiota transplantation':ab,ti OR 'washed fecal bacteria transplantation':ab,ti) AND ('randomized controlled trail':ab,ti OR 'controlled clinical trail':ab,ti OR 'random allocation':ab,ti OR 'double blind':ab,ti OR 'single blind':ab,ti OR placebo:ab,ti OR randomly:ab,ti OR randomized:ab,ti OR 'clinical trial*':ab,ti OR trial*:ab,ti OR rct:ab,ti OR random:ab,ti OR case:ab,ti OR 'case report':ab,ti OR report:ab,ti OR 'retrospective data':ab,ti OR 'retrospective study':ab,ti OR retrospective:ab,ti OR 'observational study':ab,ti OR 'clinical study':ab,ti OR 'case-control studies':ab,ti OR 'open label study':ab,ti) |
| --- | --- |
| Cochrane  Library | (((Autism Spectrum Disorder OR Autism Spectrum Disorders OR Autistic Spectrum Disorder OR Autistic Spectrum Disorders OR Disorder, Autistic Spectrum OR Asperger syndrome OR Syndrome, Asperger OR Asperger's Disease OR Asperger's Diseases OR asperger Disease OR Disease, Asperger's OR Diseases, Asperger's OR Asperger Disease OR Asperger Diseases OR Disease, Asperger OR Diseases, Asperger OR Asperger Disorder OR Asperger Disorders OR Disorder, Asperger OR Disorders, Asperger OR Asperger's Disorder OR asperger Disorder OR Disorder, Asperger's OR Asperger's Syndrome OR asperger Syndrome OR Syndrome, Asperger's OR autistic disorder OR Disorder, Autistic OR Disorders, Autistic OR kenner's Syndrome OR kenner Syndrome OR kanters Syndrome OR Autism, Infantile OR Infantile Autism OR Autism OR Autism, Early Infantile OR Early Infantile Autism OR Infantile Autism, Early):ti,ab,kw) OR (MeSH descriptor: [Autism Spectrum Disorder] explode all trees)) AND ((Transplant, Fecal OR Microbiota Transplants, Fecal OR Transplant, Fecal Microbiota OR Transplants, Fecal Microbiota OR Fecal Microbiome Transplantation OR Fecal Microbiome Transplantations OR Microbiome Transplantation, Fecal OR Microbiome Transplantations, Fecal OR Transplantation, Fecal Microbiome OR Transplantations, Fecal Microbiome OR Fecal Transplant OR Fecal Transplants OR Transplant, Fecal OR Transplants, Fecal OR Donor Feces Infusion OR Donor Feces Infusions OR Feces Infusion, Donor OR Feces Infusions, Donor OR Infusion, Donor Feces OR Infusions, Donor Feces OR Intestinal Microbiome Transplant OR Intestinal Microbiome Transplants OR Microbiome Transplant, Intestinal OR Microbiome Transplants, Intestinal OR Transplant, Intestinal Microbiome OR Transplants, Intestinal Microbiome OR Intestinal Microbiota Transfer OR Intestinal Microbiota Transfers OR Microbiota Transfer, Intestinal OR Microbiota Transfers, Intestinal OR Transfer, Intestinal Microbiota OR Transfers, Intestinal Microbiota OR Intestinal Microbiota Transplantation OR Intestinal Microbiota Transplantations OR Microbiota Transplantation, Intestinal OR Microbiota Transplantations, Intestinal OR Transplantation, Intestinal Microbiota OR Transplantations, Intestinal Microbiota OR Intestinal Microbiome Transplantation OR Intestinal Microbiome Transplantations OR Microbiome Transplantation, Intestinal OR Microbiome Transplantations, Intestinal OR Transplantation, Intestinal Microbiome OR Transplantations, Intestinal Microbiome OR Intestinal Microbiota Transplant OR Intestinal Microbiota Transplants OR Microbiota Transplant, Intestinal OR microbiota OR Transplants, Intestinal OR Transplant, Intestinal Microbiota OR Transplants, Intestinal Microbiota OR Intestinal Microbiome Transfer OR Intestinal Microbiome Transfers OR Microbiome Transfer, Intestinal OR Microbiome Transfers, Intestinal OR Transfer, Intestinal Microbiome OR Transfers, Intestinal Microbiome OR Fecal Microbiota Transfer OR Fecal Microbiota Transfers OR Microbiota Transfer, Fecal OR Microbiota Transfers, Fecal OR Transfer, Fecal Microbiota OR Transfers, Fecal Microbiota OR Fecal Transplantation OR Fecal Transplantations OR Transplantation, Fecal OR Transplantations, Fecal OR Washed Microbiota Transplantation OR Washed Fecal Bacteria Transplantation):ti,ab,kw) OR (MeSH descriptor: [Fecal Microbiota Transplantation] explode all trees)) |
| PubMed | (((((((((((((((((((((((Randomized controlled trail[Title/Abstract]) ) OR (controlled clinical trail[Title/Abstract])) OR (random allocation[Title/Abstract])) OR (double-blind[Title/Abstract])) OR (single-blind[Title/Abstract])) OR (Placebo[Title/Abstract])) OR (randomly[Title/Abstract])) OR (randomized[Title/Abstract])) OR (clinical trial*[Title/Abstract])) OR (trial*[Title/Abstract])) OR (RCT[Title/Abstract])) OR (random[Title/Abstract])) OR (case[Title/Abstract])) OR (case report[Title/Abstract])) OR (report[Title/Abstract])) OR (Retrospective data[Title/Abstract])) OR (retrospective study[Title/Abstract])) OR (retrospective[Title/Abstract])) OR (observational study[Title/Abstract])) OR (Clinical Study[Title/Abstract])) OR (Case-Control Studies[Title/Abstract])) OR (open label study[Title/Abstract]))AND ((((((((((((((((((((((((((((((((((((((("Autism Spectrum Disorder"[Mesh]) OR (Autism Spectrum Disorder[Title/Abstract])) OR (Autism Spectrum Disorders[Title/Abstract])) OR (Autistic Spectrum Disorder[Title/Abstract])) OR (Autistic Spectrum Disorders[Title/Abstract])) OR (Disorder, Autistic Spectrum[Title/Abstract])) OR (Syndrome, Asperger[Title/Abstract])) OR (Asperger's Disease[Title/Abstract])) OR (Asperger's Diseases[Title/Abstract])) OR (asperger Disease[Title/Abstract])) OR (Disease, Asperger's[Title/Abstract])) OR (Diseases, Asperger's[Title/Abstract])) OR (Asperger Disease[Title/Abstract])) OR (Asperger Diseases[Title/Abstract])) OR (Disease, Asperger[Title/Abstract])) OR (Diseases, Asperger[Title/Abstract])) OR (Asperger Disorder[Title/Abstract])) OR (Asperger Disorders[Title/Abstract])) OR (Disorder, Asperger[Title/Abstract])) OR (Disorders, Asperger[Title/Abstract])) OR (Asperger's Disorder[Title/Abstract])) OR (asperger Disorder[Title/Abstract])) OR (Disorder, Asperger's[Title/Abstract])) OR (Asperger's Syndrome[Title/Abstract])) OR (asperger Syndrome[Title/Abstract])) OR (Syndrome, Asperger's[Title/Abstract])) OR (autistic disorder[Title/Abstract])) OR (Disorder, Autistic[Title/Abstract])) OR (Disorders, Autistic[Title/Abstract])) OR (kenner's Syndrome[Title/Abstract])) OR (kenner Syndrome[Title/Abstract])) OR (kanters Syndrome[Title/Abstract])) OR (Autism, Infantile[Title/Abstract])) OR (Infantile Autism[Title/Abstract])) OR (Autism[Title/Abstract])) OR (Autism, Early Infantile[Title/Abstract])) OR (Early Infantile Autism[Title/Abstract])) OR (Infantile Autism, Early[Title/Abstract])) AND ((((((((((((((((((((((((((((((((((((((((((((((((((((((((((((((((((((((((((((("Fecal Microbiota Transplantation"[Mesh]) ) OR (Fecal Microbiota Transplantation[Title/Abstract])) OR (Fecal Microbiota Transplantations[Title/Abstract])) OR (Microbiota Transplantation, Fecal[Title/Abstract])) OR (Microbiota Transplantations, Fecal[Title/Abstract])) OR (Transplantation, Fecal Microbiota[Title/Abstract])) OR (Transplantations, Fecal Microbiota[Title/Abstract])) OR (Fecal Microbiota Transplant[Title/Abstract])) OR (Fecal Microbiota Transplants[Title/Abstract])) OR (Microbiota Transplant, Fecal[Title/Abstract])) OR (Microbiota Transplants, Fecal[Title/Abstract])) OR (Transplant, Fecal Microbiota[Title/Abstract])) OR (Transplants, Fecal Microbiota[Title/Abstract])) OR (Fecal Microbiome Transplantation[Title/Abstract])) OR (Fecal Microbiome Transplantations[Title/Abstract])) OR (Microbiome Transplantation, Fecal[Title/Abstract])) OR (Microbiome Transplantations, Fecal[Title/Abstract])) OR (Transplantation, Fecal Microbiome[Title/Abstract])) OR (Transplantations, Fecal Microbiome[Title/Abstract])) OR (Fecal Transplant[Title/Abstract])) OR (Fecal Transplants[Title/Abstract])) OR (Transplant, Fecal[Title/Abstract])) OR (Transplants, Fecal[Title/Abstract])) OR (Donor Feces Infusion[Title/Abstract])) OR (Donor Feces Infusions[Title/Abstract])) OR (Feces Infusion, Donor[Title/Abstract])) OR (Feces Infusions, Donor[Title/Abstract])) OR (Infusion, Donor Feces[Title/Abstract])) OR (Infusions, Donor Feces[Title/Abstract])) OR (Intestinal Microbiome Transplant[Title/Abstract])) OR (Intestinal Microbiome Transplants[Title/Abstract])) OR (Microbiome Transplant, Intestinal[Title/Abstract])) OR (Microbiome Transplants, Intestinal[Title/Abstract])) OR (Transplant, Intestinal Microbiome[Title/Abstract])) OR (Transplants, Intestinal Microbiome[Title/Abstract])) OR (Intestinal Microbiota Transfer[Title/Abstract])) OR (Intestinal Microbiota Transfers[Title/Abstract])) OR (Microbiota Transfer, Intestinal[Title/Abstract])) OR (Microbiota Transfers, Intestinal[Title/Abstract])) OR (Transfer, Intestinal Microbiota[Title/Abstract])) OR (Transfers, Intestinal Microbiota[Title/Abstract])) OR (Intestinal Microbiota Transplantation[Title/Abstract])) OR (Intestinal Microbiota Transplantations[Title/Abstract])) OR (Microbiota Transplantation, Intestinal[Title/Abstract])) OR (Microbiota Transplantations, Intestinal[Title/Abstract])) OR (Transplantation, Intestinal Microbiota[Title/Abstract])) OR (Transplantations, Intestinal Microbiota[Title/Abstract])) OR (Intestinal Microbiome Transplantation[Title/Abstract])) OR (Intestinal Microbiome Transplantations[Title/Abstract])) OR (Microbiome Transplantation, Intestinal[Title/Abstract])) OR (Microbiome Transplantations, Intestinal[Title/Abstract])) OR (Transplantation, Intestinal Microbiome[Title/Abstract])) OR (Transplantations, Intestinal Microbiome[Title/Abstract])) OR (Intestinal Microbiota Transplant[Title/Abstract])) OR (Intestinal Microbiota Transplants[Title/Abstract])) OR (Microbiota Transplant, Intestinal[Title/Abstract])) OR (microbiota[Title/Abstract])) OR (Transplants, Intestinal[Title/Abstract])) OR (Transplant, Intestinal Microbiota[Title/Abstract])) OR (Transplants, Intestinal Microbiota[Title/Abstract])) OR (Intestinal Microbiome Transfer[Title/Abstract])) OR (Intestinal Microbiome Transfers[Title/Abstract])) OR (Microbiome Transfer, Intestinal[Title/Abstract])) OR (Microbiome Transfers, Intestinal[Title/Abstract])) OR (Transfer, Intestinal Microbiome[Title/Abstract])) OR (Transfers, Intestinal Microbiome[Title/Abstract])) OR (Fecal Microbiota Transfer[Title/Abstract])) OR (Fecal Microbiota Transfers[Title/Abstract])) OR (Microbiota Transfer, Fecal[Title/Abstract])) OR (Microbiota Transfers, Fecal[Title/Abstract])) OR (Transfer, Fecal Microbiota[Title/Abstract])) OR (Transfers, Fecal Microbiota[Title/Abstract])) OR (Fecal Transplantation[Title/Abstract])) OR (Fecal Transplantations[Title/Abstract])) OR (Transplantation, Fecal[Title/Abstract])) OR (Transplantations, Fecal[Title/Abstract]))) |
| Web of  Science | TS=(Autism Spectrum Disorder OR Autism Spectrum Disorders OR Autistic Spectrum Disorder OR Autistic Spectrum Disorders OR Disorder, Autistic Spectrum OR Asperger syndrome OR Syndrome, Asperger OR Asperger's Disease OR Asperger's Diseases OR asperger Disease OR Disease, Asperger's OR Diseases, Asperger's OR Asperger Disease OR Asperger Diseases OR Disease, Asperger OR Diseases, Asperger OR Asperger Disorder OR Asperger Disorders OR Disorder, Asperger OR Disorders, Asperger OR Asperger's Disorder OR asperger Disorder OR Disorder, Asperger's OR Asperger's Syndrome OR asperger Syndrome OR Syndrome, Asperger's OR autistic disorder OR Disorder, Autistic OR Disorders, Autistic OR kenner's Syndrome OR kenner Syndrome OR kanners Syndrome OR Autism, Infantile OR Infantile Autism OR Autism OR Autism, Early Infantile OR Early Infantile Autism OR Infantile Autism, Early) AND TS=(Fecal Microbiota Transplantation OR Fecal Microbiota Transplantations OR Microbiota Transplantation, Fecal OR Microbiota Transplantations, Fecal OR Transplantation, Fecal Microbiota OR Transplantations, Fecal Microbiota OR Fecal Microbiota Transplant OR Fecal Microbiota Transplants OR Microbiota Transplant, Fecal OR Microbiota Transplants, Fecal OR Transplant, Fecal Microbiota OR Transplants, Fecal Microbiota OR Fecal Microbiome Transplantation OR Fecal Microbiome Transplantations OR Microbiome Transplantation, Fecal OR Microbiome Transplantations, Fecal OR Transplantation, Fecal Microbiome OR Transplantations, Fecal Microbiome OR Fecal Transplant OR Fecal Transplants OR Transplant, Fecal OR Transplants, Fecal OR Donor Feces Infusion OR Donor Feces Infusions OR Feces Infusion, Donor OR Feces Infusions, Donor OR Infusion, Donor Feces OR Infusions, Donor Feces OR Intestinal Microbiome Transplant OR Intestinal Microbiome Transplants OR Microbiome Transplant, Intestinal OR Microbiome Transplants, Intestinal OR Transplant, Intestinal Microbiome OR Transplants, Intestinal Microbiome OR Intestinal Microbiota Transfer OR Intestinal Microbiota Transfers OR Microbiota Transfer, Intestinal OR Microbiota Transfers, Intestinal OR Transfer, Intestinal Microbiota OR Transfers, Intestinal Microbiota OR Intestinal Microbiota Transplantation OR Intestinal Microbiota Transplantations OR Microbiota Transplantation, Intestinal OR Microbiota Transplantations, Intestinal OR Transplantation, Intestinal Microbiota OR Transplantations, Intestinal Microbiota OR Intestinal Microbiome Transplantation OR Intestinal Microbiome Transplantations OR Microbiome Transplantation, Intestinal OR Microbiome Transplantations, Intestinal OR Transplantation, Intestinal Microbiome OR Transplantations, Intestinal Microbiome OR Intestinal Microbiota Transplant OR Intestinal Microbiota Transplants OR Microbiota Transplant, Intestinal OR microbiota OR Transplants, Intestinal OR Transplant, Intestinal Microbiota OR Transplants, Intestinal Microbiota OR Intestinal Microbiome Transfer OR Intestinal Microbiome Transfers OR Microbiome Transfer, Intestinal OR Microbiome Transfers, Intestinal OR Transfer, Intestinal Microbiome OR Transfers, Intestinal Microbiome OR Fecal Microbiota Transfer OR Fecal Microbiota Transfers OR Microbiota Transfer, Fecal OR Microbiota Transfers, Fecal OR Transfer, Fecal Microbiota OR Transfers, Fecal Microbiota OR Fecal Transplantation OR Fecal Transplantations OR Transplantation, Fecal OR Transplantations, Fecal OR Washed Microbiota Transplantation OR Washed Fecal Bacteria Transplantation AND TS=(Randomized controlled trial OR controlled clinical trial OR random allocation OR double-blind OR single-blind OR Placebo OR randomly OR randomized OR clinical trial* OR trial* OR RCT OR random OR case OR case report OR report OR Retrospective data OR retrospective study OR retrospective OR observational study OR Clinical Study OR Case-Control Studies OR open-label study ) |

**
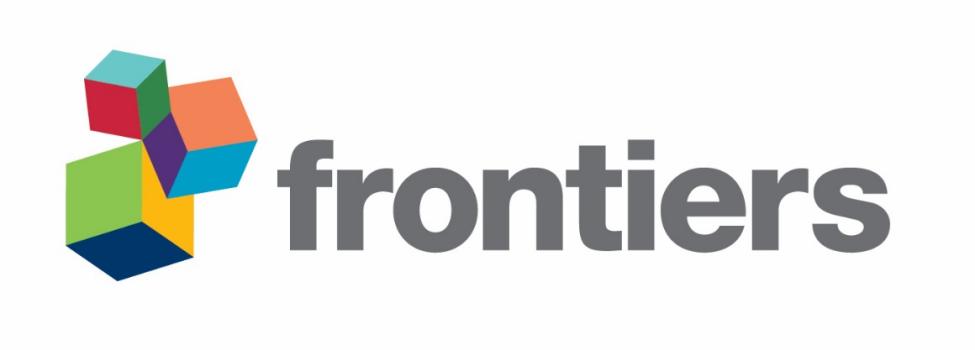
**
